# LncRNA MIR4435‐2HG potentiates the proliferation and invasion of glioblastoma cells via modulating miR‐1224‐5p/TGFBR2 axis

**DOI:** 10.1111/jcmm.15280

**Published:** 2020-04-22

**Authors:** Hongchao Xu, Beilin Zhang, Yinggui Yang, Zihuang Li, Pan Zhao, Weiqing Wu, Huirong Zhang, Jie Mao

**Affiliations:** ^1^ Clinical Medical Research Center The First Affiliated Hospital of Southern University Shenzhen People's Hospital The Second Clinical Medical College of Jinan University Shenzhen China; ^2^ Department of Neurology The First Teaching Hospital of Jilin University Changchun China; ^3^ Shenzhen Key Laboratory of Viral Oncology the Clinical Innovation& Research Center (CIRC), Shenzhen People's Hospital The Second Clinical Medical College of Jinan University The First Affiliated Hospital of Southern University Shenzhen China; ^4^ Department of Physical Examination The First Affiliated Hospital of Southern University Shenzhen People's Hospital The Second Clinical Medical College of Jinan University Shenzhen China; ^5^ Department of Health management The First Affiliated Hospital of Southern University Shenzhen People's Hospital The Second Clinical Medical College of Jinan University Shenzhen China; ^6^ Department of Neurosurgery Shenzhen Hospital Southern Medical University Shenzhen China

**Keywords:** glioblastoma, miR‐1224‐5p, MIR4435‐2HG, proliferation and invasion, TGFBR2

## Abstract

Glioblastoma (GBM) belongs to the high‐grade (IV) gliomas with extremely poor prognosis. Accumulating evidence uncovered the key roles of long non‐coding RNAs (lncRNAs) in GBM development. This study aimed to determine the biological actions and the clinical relevance of lncRNA MIR4435‐2 Host Gene (MIR4435‐2HG) in GBM. Data from GEPIA database showed that MIR4435‐2HG was up‐regulated in GBM tissues and high expression of MIR4435‐2HG correlated with shorter overall survival of GBM patients. Further experimental assays verified the up‐regulation of MIR4435‐2HG in GBM tissues and cell lines. In vitro cell studies and in vivo animal studies showed that knockdown of MIR4435‐2HG resulted in the inhibition of GBM cell proliferation and invasion and in vivo tumour growth, while MIR4435‐2HG overexpression driven GBM progression. Furthermore, MIR44435‐2HG was found to sponge miR‐1224‐5p and suppress miR‐1224‐5p expression; overexpression of miR‐1224‐5p attenuated the enhancement in GBM cell proliferation and invasion induced by MIR4435‐2HG overexpression. In a subsequent study, miR‐1224‐5p was found to target transforming growth factor‐beta receptor type 2 (TGFBR2) and repressed TGFBR2 expression, and in vitro assays showed that miR‐1224‐5p exerted tumour‐suppressive effects via targeting TGFBR2. More importantly, TGFRB2 knockdown antagonized hyper‐proliferation and invasion of GBM cells with MIR4435‐2HG overexpression. Clinically, the down‐regulation of miR‐1224‐5p and up‐regulation of TGFBR2 were verified in the GBM clinical samples. Taken together, the present study suggests the oncogenic role of MIR4435‐2HG in GBM and underlies the key function of MIR4435‐2HG‐driven GBM progression via targeting miR‐1224‐5p/TGFBR2 axis.

## 1 INTRODUCTION

Glioma is one of the most common types of brain tumours and belongs to astrocytic tumours.^1^ Glioma can be divided into four grades (I, II, III and IV) based on the World Health Organization (WHO) classification.^2^ Low‐grade glioma belongs to WHO grades I and II, while high‐grade glioma belongs to WHO grades III and IV.^2^ Glioblastoma belongs to the WHO IV glioma.^2^ Patients with glioblastoma without developing from low‐grade glioma were diagnosed as primary glioblastoma; while secondary glioblastoma was developed from low‐grade glioma.^3^, ^4^ Though various therapeutic strategies including surgery, chemotherapy and radiotherapy have been developed, the heterogeneity of glioblastoma cells makes the tumour cells be less sensitive to chemo/radiotherapy.^5^, ^6^ Thus, the outcomes of the current treatments for glioblastoma are un‐satisfying and the prognosis is extremely poor in the patients with glioblastomas.^7^ Therefore, there is an urgent need for the scientific community to develop novel therapies for the treatment of glioblastoma.

Long non‐coding RNAs (lncRNAs) belong to a class of the non‐coding RNAs, and lncRNAs are longer than 200 nucleotide base pairs in length and are lack of capacity for coding proteins.^8^ According to the genomic studies, the human genome has more than 50,000 lnRNA genes, and the functional roles of these lncRNAs are poorly studied.^9^ Up to date, lncRNAs have been elucidated for their roles in various aspects of cellular functions including apoptosis, proliferation, metastasis, and stemness, which are closely related to pathogenesis of various diseases including glioblastoma.^10^, ^11^, ^12^ For examples, Voce et al, found that lncRNA metastasis‐associated lung adenocarcinoma transcript‐1 (MALAT1) was a target for GBM chemo‐sensitization to temozolomide, though the up‐regulation of MALAT1 in GBM tissues was not a prognostic factor for the overall survival of GBM patients.^13^ Wu et al, showed that lncRNA MIR155 host gene/miR‐185/annexin A2 loop had a regulatory role in the GBM progression, which underlies the importance of lncRNA MIR155 host gene in the GBM pathophysiology.^14^ Liu et al, found that LINC00470 epigenetically regulated the expression of extracellular leucine rich repeat and fibronectin type III domain containing 2 to distract cell autophagy in GBM.^15^ Recent studies suggest the lncRNA MIR4435‐2 Host Gene (MIR4435‐2HG) may involve in the regulation of brain tumour progression. Specifically, MIR4435‐2HG encodes a nearly identical hairpin of LINC00152, while LINC00152 was associated with aggressive tumours and the enhanced proliferation of GBM cells.^16^ In addition, MIR4435‐2HG exerted the oncogenic functions in other types of malignant tumours including hepatocellular carcinoma,^17^ colorectal cancer,^18^ gastric cancer ^19^ and lung cancer.^20^ To date, the underlying mechanisms of MIR4435‐2HG in GBM progression has yet to be explored.

Here, we showed that MIR4435‐2HG is clinically relevant in GBM, as MIR4435‐2HG is up‐regulated in GBM tissues and high expression of MIR4435‐2HG is associated with poor overall survival rate of GBM patients. Furthermore, MIR4435‐2HG knockdown leads to the inhibition of GBM cell proliferation and invasion; while MIR4435‐2HG overexpression drives the GBM progression. The mechanistic studies revealed that MIR4435‐2HG exerts its actions via targeting miR‐1224‐5p/ transforming growth factor‐beta receptor type 2 (TGFBR2) axis in GBM. Together, the present study underlies the key function of MIR4435‐2HG‐driven GBM progression and brings forth MIR4435‐2HG as a therapeutic target for this malignant tumour.

## 2 MATERIALS AND METHODS

### 2.1 Clinical sample collection

A total of 40 patients diagnosed with GBM were recruited in this study, and this study was approved by the Ethics Committee of Shenzhen People's Hospital, and all the patients signed the written informed consent. The GBM tissues as well as the adjacent normal tissues were collected from these patients who underwent surgical resection for brain tumours between January 2016 and December 2018 in the Shenzhen People's Hospital. The GBM patients had not received any chemotherapy or radiotherapy before surgery. All the clinical samples were collected into liquid nitrogen and stored in −80°C for further examination.

### 2.2 Cell culture

The normal human astrocytes (NHAs) were obtained from Lonza and were cultured in ABM growth medium (ABM^™^, Lonza). The GBM cell lines (LN229, U87MG, U87 and U251) were all obtained from ATCC and were cultured in DMEM (ATCC^®^ 30‐2002^™^) supplemented with 10% fetal bovine serum (FBS; ATCC^®^ 30‐2020^™^). All the NHAs and GBM cells were maintained in a humidified incubator with 5% CO_2_ at 37°C.

### 2.3 Small interfering RNAs (siRNAs), plasmids, microRNAs (miRNAs) and cell transfections

The siRNAs respectively targeted MIR4435‐2HG (designated as siRNA#1 and #2) and TGFBR2 (designated as si‐TGFBR2) were designed and synthesized by RiboBio, and the scrambled siRNAs serve as negative controls (NCs; Ribobio). The pcDNA3.1 was utilized to construct the MIR4435‐2HG and TGFBR2 overexpression vectors (named as pcDNA3.1‐MIR4435‐2HG and pcDNA3.1‐TGFBR2, respectively), and pcDNA3.1 was used as the NCs (GenePharma). The miRNAs including miR‐1224‐5p mimics and the NC for mimics (mimics NC) were purchased from Ribobio. The short‐hairpin RNAs (shRNAs) that target MIR4435‐2HG as well as the scrambled shRNA were packaged in lentivirus and were infected into GBM cells to generate the stable GBM cell lines for MIR4435‐2HG knockdown. All the GBM cell transfections were conducted using Lipofectamine 2000 reagent (ThermoFisher Scientific).

### 2.4 RNA extraction, cDNA synthesis and quantitative real‐time PCR

Total RNA from clinical samples, tumour tissues and culture cells was purified using the MiniBest Universal RNA extraction kit (Takara) by following the manufacturer's protocol. Briefly, mRNA was reversely transcribed into cDNA using Reverse Transcription Reagents (Takara), while TaqMan miRNA Reverse Transcription Kit (Applied Biosystems) was utilized to perform miRNA reverse transcription. Real‐time PCR was performed using SYBR Select Master Mix kit (Roche) on ABI7900 PCR detection systems (Applied Biosystems). MIR4435‐2HG and TGFBR2 expression were normalized based on the glyceraldehyde 3‐phosphate dehydrogenase (GAPDH) expression; while miR‐1224‐5p expression was normalized based on U6 expression. Comparative Ct method was used to determine the gene expression levels. The primers were showing below: MIR4435‐2HG, F: 5′‐ACTCTGAAACTACCCGGCTC‐3′ and R: 5′‐GTCAACGCGGAAAAGACACT‐3′; miR‐1224‐5p, F: 5′‐ACACTCCAGCTGGGGTGAGGACTGGGG‐3’ and R: 5′‐TGGTGTCGTGGAGTCG‐3′; TGFBR2, F: 5′‐ACTGCCCATCCACTGAGACAT‐3′ and R: 5′‐CCATACAGCCACACAGACTTCC‐3′; U6, F: 5′‐CTCGCTTCGGCAGCACA‐3′ and R: 5′‐AACGCTTCACGAATTTGCGT‐3′; GAPDH, F: 5′‐GGTGAAGGTCGGTGTGAACG‐3′ and R: 5′‐ CTCGCTCCTGGAAGATGGTG‐3′.

### 2.5 Cell proliferation assay

The cell proliferative ability of U87 and U251 cells were determined by the Cell Counting Kit‐8 (CCK‐8; Beyotime) assay. In brief, the transfected U87 and U251 cells were seeded onto the 96‐well plates, and after a further culture for indicated time durations (0, 24, 48 and 72 hours), the transfected U87 and U251 cells were subjected to incubation with 10 μL CCK‐8 solution for 2 hours at 37°C. The optical density was examined at 450 nm wavelength using a Microplate Reader (Bio‐Rad) to determine cell proliferative index.

### 2.6 Colony formation assay

The cell growth of U87 and U251 cells were assessed using colony formation assay. In brief, the transfected U87 and U251 cells were collected and inoculated into the 6‐well plates, and GBM cells were then cultured for 10 days. The cultured medium was refreshed every 3 days. At 10 d after inoculation, the colonies were visualized by staining the GBM cells with 0.1% crystal violet, and the number of colonies was counted with a light microscope.

### 2.7 Cell invasion assay

Cell invasive ability was determined using the transwell invasion assay. In brief, the transwell inserts with 8 µm pore size filters (Millipore) were coated with Matrigel (Sigma), and the transfected U87 and U251 cells were seeded onto the upper chamber with transwell inserts. The upper chamber was filled with serum‐free medium, while lower chamber was filled with medium containing 20% FBS. The transfected U87 and U251 cells were cultured for 24 hours, after that, the cells invaded into the lower filter membranes were fixed with 100% methanol and stained with 0.1% crystal violet for the quantification of the invasive cell number.

### 2.8 Cell apoptosis assays

Cell apoptotic rates of the transfected U87 and U251 cells were determined on a flow cytometer using the Annexin V‐FITC/propidium iodide Cell Apoptosis Detection kit (Thermo Fisher Scientific) by following the manufacturer's protocol.

Capase‐3 activities of the transfected U87 and U251 cells were evaluated using the Caspase‐3 Activity Assay kit (Thermo Fisher Scientific) by following the manufacturer's protocol.

### 2.9 In vivo tumorigenicity assay

The male athymic BALB/c mice (4‐5 weeks old) were purchased from Vital River Laboratories. All the animal experimental procedures were approved by the Animal Ethics Committee of Shenzhen People's Hospital. U87 cells with stable MIR4435‐2HG overexpression or knockdown were injected subcutaneously into the right flank of the animals (each group had 5 mice). After injection, the tumour volume was measured from day 7 to day 35 with a 7‐day internal. The tumour volume was measured using the following formula: volume = length × width × width/2. The mice were euthanized at 35 days after cell injection, and the tumours were taken out for further determinations.

### 2.10 Western blot assay

Proteins from transfected cells were extracted using RIPA buffer (Sigma) supplemented with Protease Inhibitor Cocktail (Roche). The protein concentrations of the lysed samples were measured using the bicinchoninic acid assay (Bio‐Rad). The proteins were electrophoresed on a 10% SDS‐PAGE and were then transferred to the polyvinylidene difluoride (PVDF) membranes. After incubating with 5% skimmed milk for 1 hour at room temperature, the PVDF membranes were probed with primary antibodies against TGFBR2 (Cell Signaling Technology) and β‐actin (Cell Signaling Technology) by a further overnight incubation at 4°C. Afterwards, the PVDF membranes were washed with phosphate buffered saline with Tween‐20 for 3 times × 5 mins and were then incubated with horseradish peroxidase‐labelled secondary antibodies (Cell Signaling Technology). The Western blot signals were visualized using the ELC Substrates (ThermoFisher Scientific).

### 2.11 Dual‐luciferase reporter assay

The DNA segments from MIR4435‐2HG and TGFBR2 3’ untranslated region (3’UTR) were amplified from human genomic DNA and were subcloned into the pmirGLO vector (Promega, Madison, USA) to construct the wild‐type (WT) luciferase reporter vectors (MIR4435‐2HG‐WT and TGFBR2 3’UTR‐WT). The respective fragments were also mutated using the Site‐Directed Mutagenesis Kit (Stratagene, San Diego, USA) to generate the mutant (Mut) reporter vectors (MIR4435‐2HG‐MUT and TGFBR2 3’UTR‐Mut). For the luciferase activity detection, U87 cells were co‐transfected with miRNAs (miR‐1224‐5p mimics or mimics NC) and the relevant luciferase reporter vectors using the Lipofectamine 2000 reagent (Invitrogen) and the luciferase activity was assessed at 48 hours after transfection using Dual‐Luciferase Report Assay System (Progema).

### 2.12 Statistical analysis

SPSS software V20.0 (IBM, Armonk, USA) and GraphPad Prims V6.0 (GraphPad Software) were used to perform data analysis. In each experiment, triplicate samples were used for experimental assays. The data were shown as mean ± standard deviation. Statistical comparison between groups were analysed using unpaired Student's *t* test or one‐way ANOVA followed with Bonferroni's multiple comparison tests. Correlation between two variables were determined using Pearson's Correlation analysis. *P* < .05 was accepted as statistical significance.

## 3 RESULTS

### 3.1 MIR4435‐2HG is up‐regulated in GBM tissues

**FIGURE 1:**
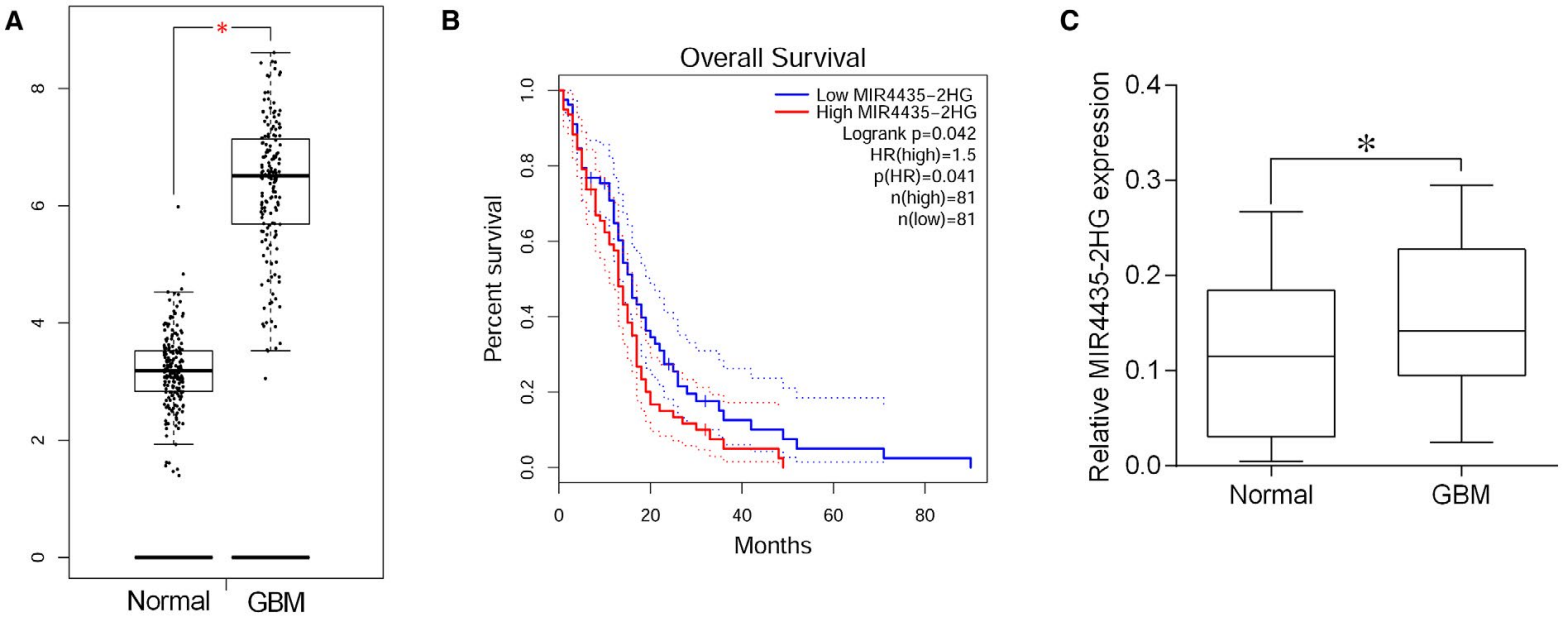
MIR4435‐2HG is up‐regulated in GBM tissues. A, MIR4435‐2HG was up‐regulated in GBM tissues compared to normal tissues as determined from GEPIA database. B, GBM patients with high expression of MIR4435‐2HG has shorter overall survival rate than that with lower expression of MIR4435‐2HG as determined from GEPIA database. C, MIR4435‐2HG in clinical samples was determined by qRT‐PCR, and MIR4435‐2HG was up‐regulated in GBM tissues from 40 patients compared to normal adjacent tissues. *P < .05

The expression of MIR4435‐2HG was first analysed from the GEPIA database (http://gepia.cancer‐pku.cn/); based on the database, the expression of MIR4435‐2HG was compared between 207 normal tissues and 163 GBM tissues. As shown in Figure 1A, MIR4435‐2HG expression was significantly up‐regulated in the GBM group compared to that in the normal group. The overall survival data from GEPIA database showed that GBM patients with high expression of MIR4435‐2HG had shorter overall survival rate than that with low expression of MIR4435‐2HG (Figure 1B). To confirm the results from GEPIA database, we analysed the expression of MIR4435‐2HG in GBM tissues and adjacent normal tissues from 40 patients using qRT‐PCR, and MIR4435‐2HG was up‐regulated in GBM tissues from 40 patients compared to normal adjacent tissues (Figure 1C).

### 3.2 *Knockdown of MIR4435‐2HG inhibited GBM cell proliferation and invasion and *in vivo* tumour growth*

**FIGURE 2:**
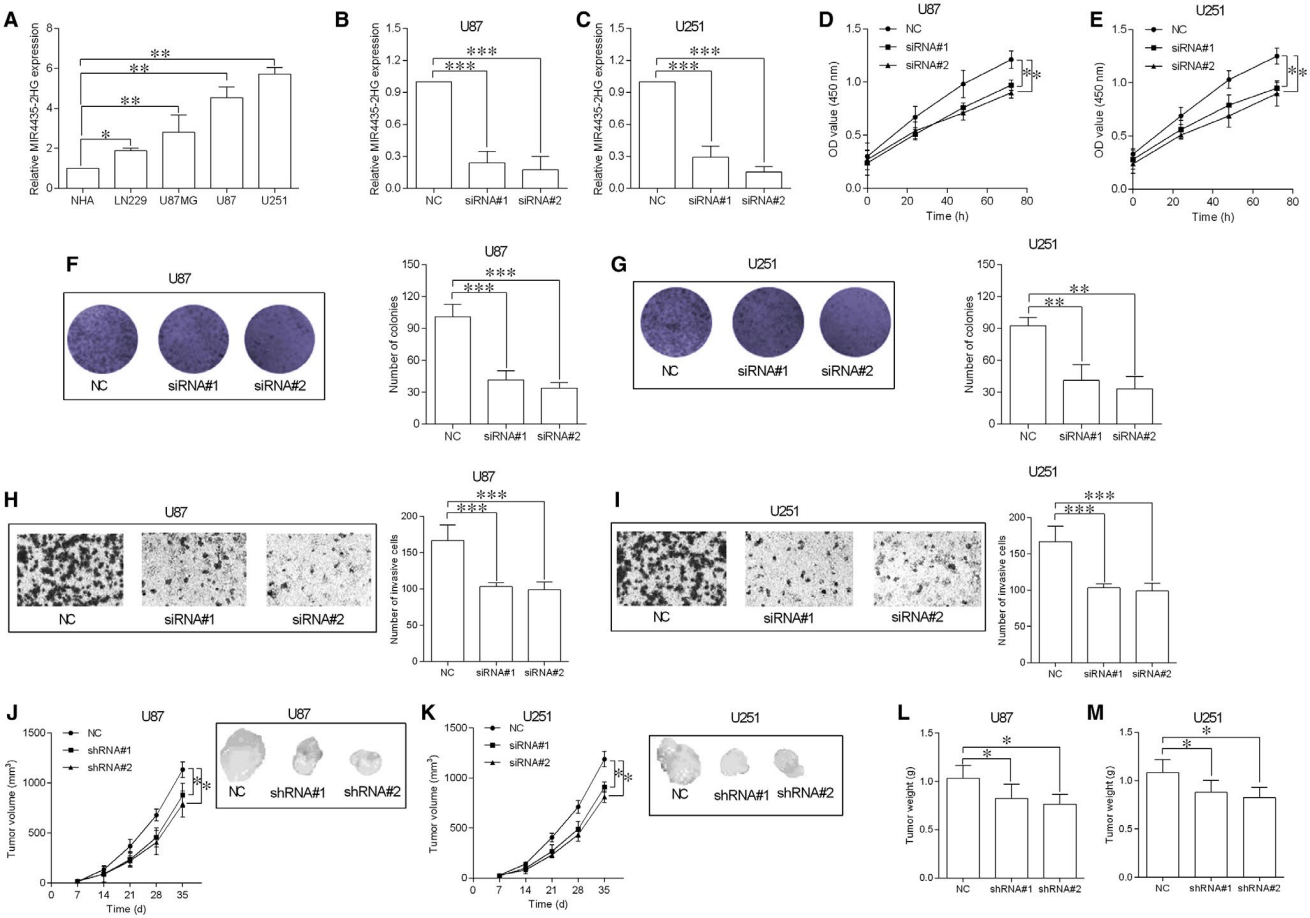
Knockdown of MIR4435‐2HG inhibited GBM cell proliferation and invasion and in vivo tumour growth. A, MIR4435‐2HG expression in normal human astrocytes (NHA) and GBM cell lines including LN229, U87MG, U87, and U251 was determined by qRT‐PCR (n = 3). B and C, qRT‐PCR showed the down‐regulation of MIR4435‐2HG expression in U87 (B) and U251 cells (C) by transfecting with MIR4435‐2HG siRNAs (siRNA#1 and siRNA#2), NC = scrambled siRNA (n = 3). D and E, CCK‐8 assay was utilized to determine the proliferative ability of the transfected U87 (D) and U251 (E) cells (n = 3). F and G, Colony formation assay was utilized to determine the cell growth of the transfected U87 (F) and U251 (G) cells (n = 3). H and I, Transwell invasion assay was utilized to assess the cell invasive ability of the transfected U87 (H) and U251 (I) cells (n = 3). J and K, In vivo tumour growth assay was used to determine the cell growth of the transfected U87 (J) and U251 (K) cells (n = 5). L and M, The weight of the dissected tumours was determined from NC (scrambled shRNA) group and shRNAs group (shRNA#1 and shRNA#2) (n = 5). *P < .05, **P < .01 and ***P < .001

The expression of MIR4435‐2HG was detected in different cell lines, and MIR4435‐2HG expression was up‐regulated in GBM cell lines including LN229, U87MG, U87 and U251 compared to NHA cells (Figure 2A). The knockdown of MIR4435‐2HG in U87 and U251 cells were detected when cells transfected with MIR4435‐2HG siRNAs (siRNA#1 and #2) (Figure 2B,C). The effects of MIR4435‐2HG knockdown on cell proliferation, growth and invasion of the transfected cells were further detected by CCK‐8, colony formation and transwell invasion assays. As shown in Figure 2D,E, MIR4435‐2HG knockdown significantly inhibited the cell proliferation of U87 and U251 cells. The number of colonies for U87 and U251 cells were obviously reduced by transfecting with MIR4435‐2HG siRNAs (Figure 2F,G). In addition, MIR4435‐2HG knockdown also increased cell apoptotic rates and caspase‐3 activities of U87 and U251 cells (Figure S1). Consistently, knockdown of MIR4435‐2HG also impaired the invasive ability of U87 and U251 cells (Figure 2H,I). In vivo xenograft nude model assessed the effects of MIR4435‐2HG knockdown on U87 and U251 in vivo tumour growth, and MIR4435‐2HG knockdown significantly suppressed the tumour volume at different time points and the weight of the dissected tumours (Figure 2J‐M).

### 3.3 *Overexpression of MIR4435‐2HG promoted GBM cell proliferation and invasion and *in vivo* tumour growth*

**FIGURE 3:**
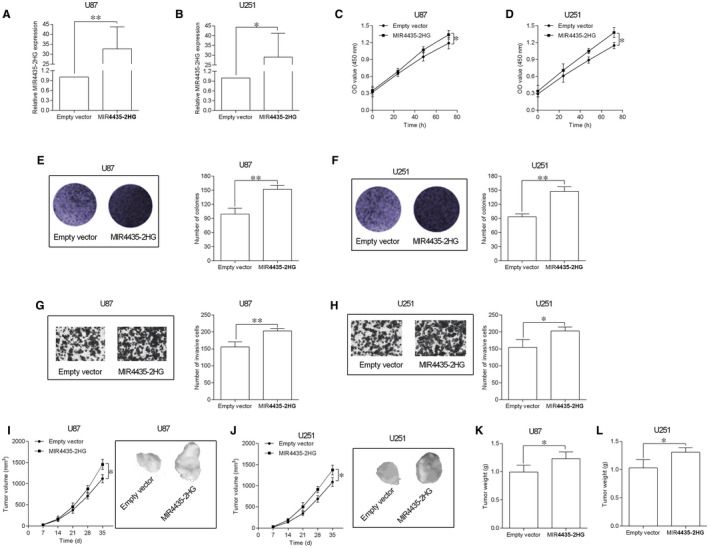
Overexpression of MIR4435‐2HG promoted GBM cell proliferation and invasion and in vivo tumour growth. A and B, qRT‐PCR showed the up‐regulation of MIR4435‐2HG expression in U87 (A) and U251 cells (B) by transfecting with pcDNA3.1‐MIR4435‐2HG; empty vector = pcDNA3.1 (n = 3). C and D, CCK‐8 assay was utilized to determine the proliferative ability of the transfected U87 (C) and U251 (D) cells (n = 3). E and F, Colony formation assay was utilized to determine the cell growth of the transfected U87 (E) and U251 (F) cells (n = 3). G and H, Transwell invasion assay was utilized to assess the cell invasive ability of the transfected U87 (G) and U251 (H) cells (n = 3). J and K, In vivo tumour growth assay was used to determine the cell growth of the transfected U87 (J) and U251 (K) cells (n = 5). L and M, The weight of the dissected tumours was determined from empty vector (pcDNA3.1) group and pcDNA3.1‐MIR4435‐2HG group (n = 5). *P < .05 and **P < .01

The MIR‐4435‐2HG overexpression in U87 and U251 cells were performed by transfecting with pcDNA3.1‐MIR4435‐2HG (Figure 3A,B). The MIR4435‐2HG overexpression effects on cell proliferation, growth and invasion of the transfected cells were determined by the same assays. MIR4435‐2HG overexpression significantly potentiated cell proliferation of U87 and U251 cells and also increased the number of colonies in U87 and U251 cells (Figure 2C‐F). In addition, MIR4435‐2HG overexpression enhanced the invasive abilities of U87 and U251 cells (Figure 3G,H). In vivo xenograft nude model assessed the effects of MIR4435‐2HG overexpression on U87 and U251 in vivo tumour growth, and MIR4435‐2HG overexpression significantly accelerated the tumour growth at different time points and increased the weight of the dissected tumours (Figure 3I‐L).

### 3.4 MIR4435‐2HG acts as a sponge for miR‐1224‐5p

**FIGURE 4:**
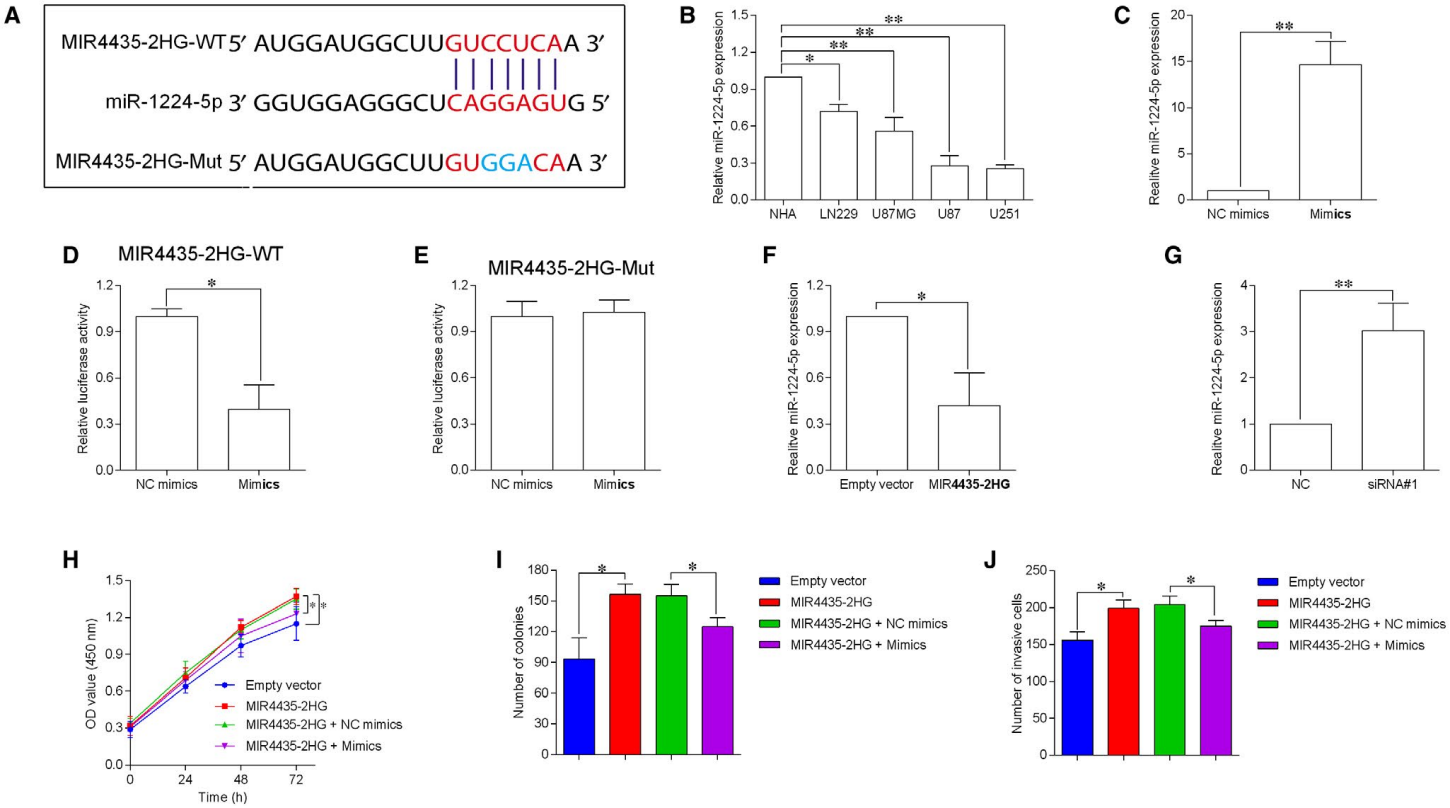
MIR4435‐2HG acts as a sponge for miR‐1224‐5p. A, MiR‐1224‐5p had a binding site for MIR4435‐2HG as predicted by starBase database. B, MiR‐1224‐5p expression in normal human astrocytes (NHA) and GBM cell lines including LN229, U87MG, U87, and U251 was determined by qRT‐PCR (n = 3). C, qRT‐PCR showed the up‐regulation of miR‐1224‐5p expression in U87 cells by transfecting with miR‐1224‐5p mimics (mimics) (n = 3). D and E, Luciferase reporter assay determined the relative luciferase activity of U87 cells by co‐transfection with miRNAs (mimics NC or mimics) and reporter vectors (MIR4435‐2HG‐WT or MIR4435‐2HG‐Mut). F, qRT‐PCR determination of miR‐1224‐5p expression in U87 cells by transfecting with pcDNA3.1 (empty vector) or pcDNA3.1‐MIR4435‐2HG. G, qRT‐PCR determination of miR‐1224‐5p expression in U87 cells by transfecting with MIR4435‐2HG siRNA (siRNA#1) or scrambled siRNA (NC). H‐J, CCK‐8, colony formation and transwell invasion assays were performed to determined cell proliferation, growth and invasion of the transfected/co‐transfected U87 cells. N = 3. *P < .05 and **P < .01

The starBase tool was utilized to predict the potential miRNAs for MIR4435‐2HG and the prediction results showed that miR‐1224‐5p had a binding site for MIR4435‐2HG (Figure 4A). The results from qRT‐PCR assay showed that miR‐1224‐5p was down‐regulated in LN229, U87MG, U87, and U251 cells compared to NHA cells (Figure 4B). The findings from the luciferase report assay showed that the luciferase activity of MIR4435‐2HG‐WT was suppressed by transfecting with miR‐1224‐5p mimics in U87 cells (Figure 4C,D), while MIR4435‐2HG‐Mut luciferase activity was unaffected by miR‐1224‐5p overexpression (Figure 4E). The further qRT‐PCR showed that miR‐1224‐5p expression was down‐regulated in U87 cells upon MIR4435‐2HG overexpression (Figure 4F); while being up‐regulated upon MIR4435‐2HG knockdown (Figure 4G). The rescue experiments were performed to examine whether MIR4435‐2HG‐induced GBM progression via targeting miR‐1224‐5p. The CCK‐8 assay revealed that miR‐1224‐5p overexpression counteracted MIR4435‐2HG overexpression‐induced an increase in U87 cell proliferation and growth (Figure 4H,I). Furthermore, miR‐1224‐5p mimics reversed the increased cell invasive number induced by MIR4435‐2HG overexpression in U87 cells (Figure 4J).

### 3.5 TGFBR2 is a direct target of miR‐1224‐5p

**FIGURE 5:**
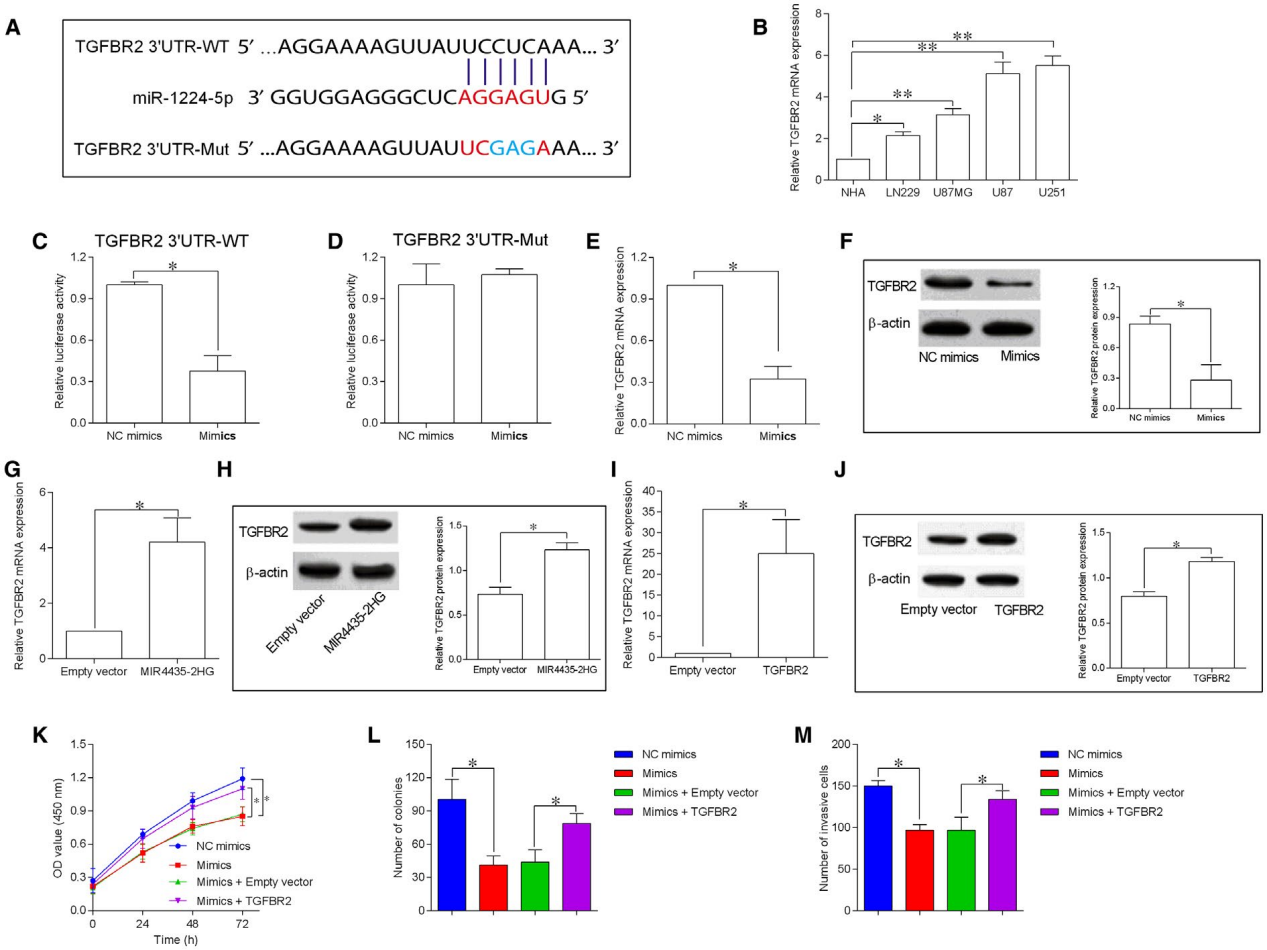
TGFBR2 is a direct target of miR‐1224‐5p. A, TGFBR2 3’UTR had a binding site for miR‐1224‐5p as predicted by starBase V3.0. B, TGFBR2 mRNA expression in normal human astrocytes (NHA) and GBM cell lines including LN229, U87MG, U87 and U251 was determined by qRT‐PCR (n = 3). C and D, Luciferase reporter assay determined the relative luciferase activity of U87 cells by co‐transfection with miRNAs (mimics NC or mimics) and reporter vectors (TGFBR2 3’UTR‐WT or TGFBR2 3’UTR‐Mut). E and F, qRT‐PCR and Western blot determination of TGFBR2 mRNA and protein expression in U87 cells by transfecting with mimics NC or miR‐1224‐5p mimics. G and H, qRT‐PCR and Western blot determination of TGFBR2 mRNA and protein expression in U87 cells by transfecting with empty vector (pcDNA3.1) or pcDNA3.1‐MIR4435‐2HG. I and J, qRT‐PCR and Western blot determination of TGFBR2 mRNA and protein expression in U87 cells by transfecting with empty vector (pcDNA3.1) or pcDNA3.1‐TGFBR2. K‐M, CCK‐8, colony formation and transwell invasion assays were performed to determined cell proliferation, growth and invasion of the transfected/co‐transfected U87 cells. N = 3. *P < .05 and **P < .01

To further predicted the targets for miR‐1224‐5p, we employed the starBase tool to predict the potential targets and found that TGFBR2 3’UTR had a binding site for miR‐1224‐5p (Figure 5A). The results from qRT‐PCR assay showed that TGFBR2 mRNA was up‐regulated in LN229, U87MG, U87, and U251 cells compared to NHA cells (Figure 5B). TGFBR2 3’UTR‐WT luciferase activity significantly supressed by miR‐1224‐5p mimics transfection in U87 cells (Figure 5C), while TGFBR2 3’UTR‐Mut luciferase activity showed no significant change (Figure 5D). To investigate the impact of miR‐1224‐5p and MIR4435‐2HG on TGFBR2 expression, we further performed qRT‐PCR assay. The TGFBR2 mRNA and protein levels were suppressed by miR‐1224‐5p up‐regulation (Figure 5E,F); while being increased by MIR4435‐2HG overexpression in U87 and U251 cells (Figure 5G,H). The rescue experiments were performed to examine whether miR‐1224‐5p regulated GBM progression via targeting TGFBR2. As shown in Figure 5I,J TGFBR2 overexpression was detected in U87 cells by transfecting with pcDNA3.1‐TGFBR2. The in vitro assay showed that miR‐1224‐5p overexpression imposed the inhibitory actions on U87 cell proliferation, growth and invasion (Figure 5K‐M), which was partially antagonized by TGFBR2 overexpression (Figure 5K‐M).

### 3.6 MIR4435‐2HG regulated proliferation, growth and invasion of U87 cells via regulating TGFBR2

**FIGURE 6:**
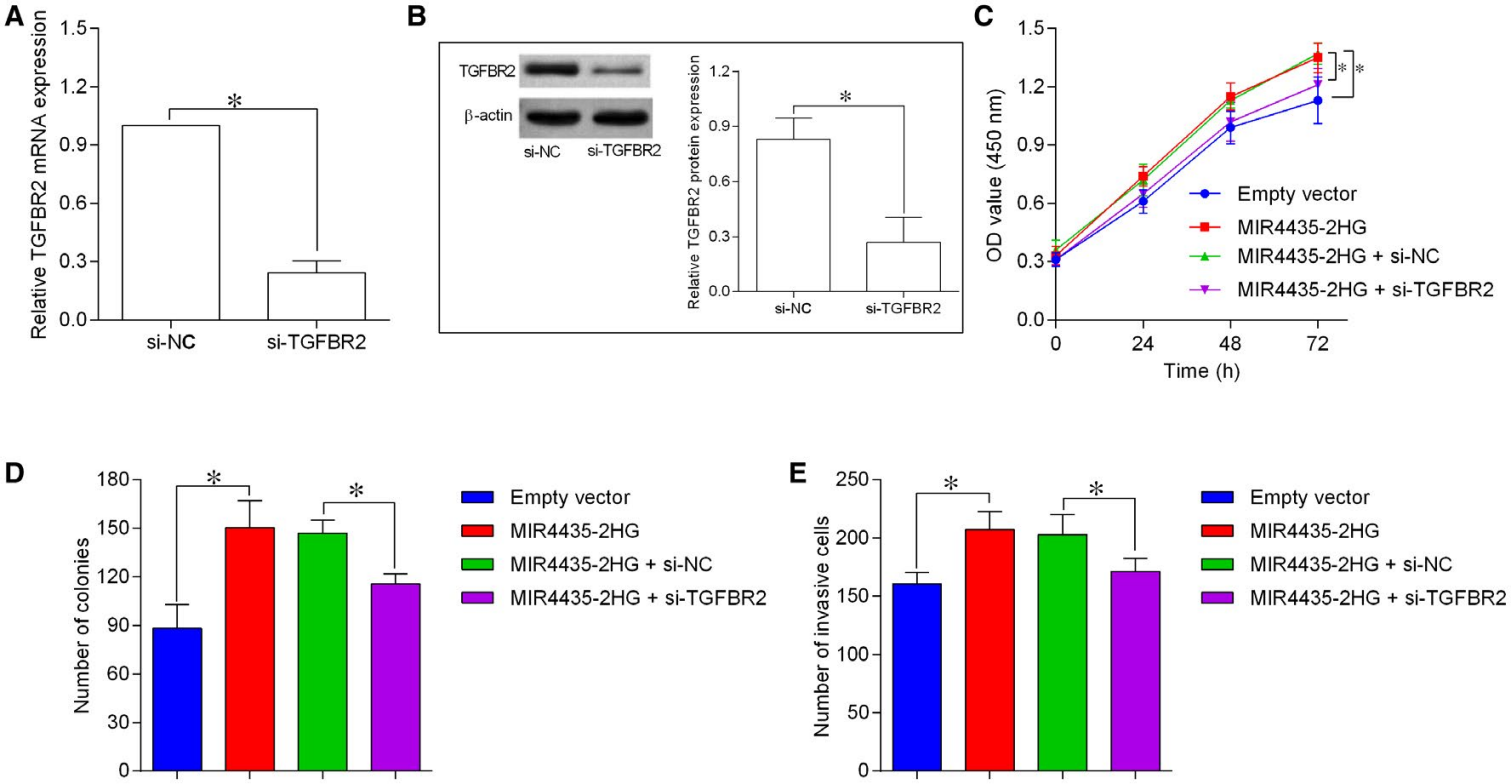
MIR4435‐2HG regulated cell proliferation, growth and invasion of U87 cells via regulating TGFBR2. A and B, qRT‐PCR and Western blot determination of TGFBR2 mRNA and protein expression in U87 cells by transfecting with TGFBR2 siRNA (si‐TGFBR2) or scrambled siRNA (si‐NC). C‐E, CCK‐8, colony formation and transwell invasion assays were performed to determined cell proliferation, growth and invasion of the transfected/co‐transfected U87 cells. N = 3. *P < .05

To determine if MIR4435‐2HG regulated GBM progression via modulating TGFBR2, we further performed the rescue experiments. The down‐regulation of TGFBR2 were detected in U87 cells with TGFBR2 siRNA transfection (Figure 6A,B). The CCK‐8 assay revealed that TGFBR2 knockdown antagonized MIR4435‐2HG overexpression‐induced an enhancement in U87 cell proliferation and growth (Figure 6C,D). Additionally, TGFBR2 inhibition reversed the increased cell invasive number induced by MIR4435‐2HG overexpression in U87 cells (Figure 6E).

### 3.7 MIR‐1224‐5p and TGFBR2 mRNA expression in clinical tissues

**FIGURE 7:**
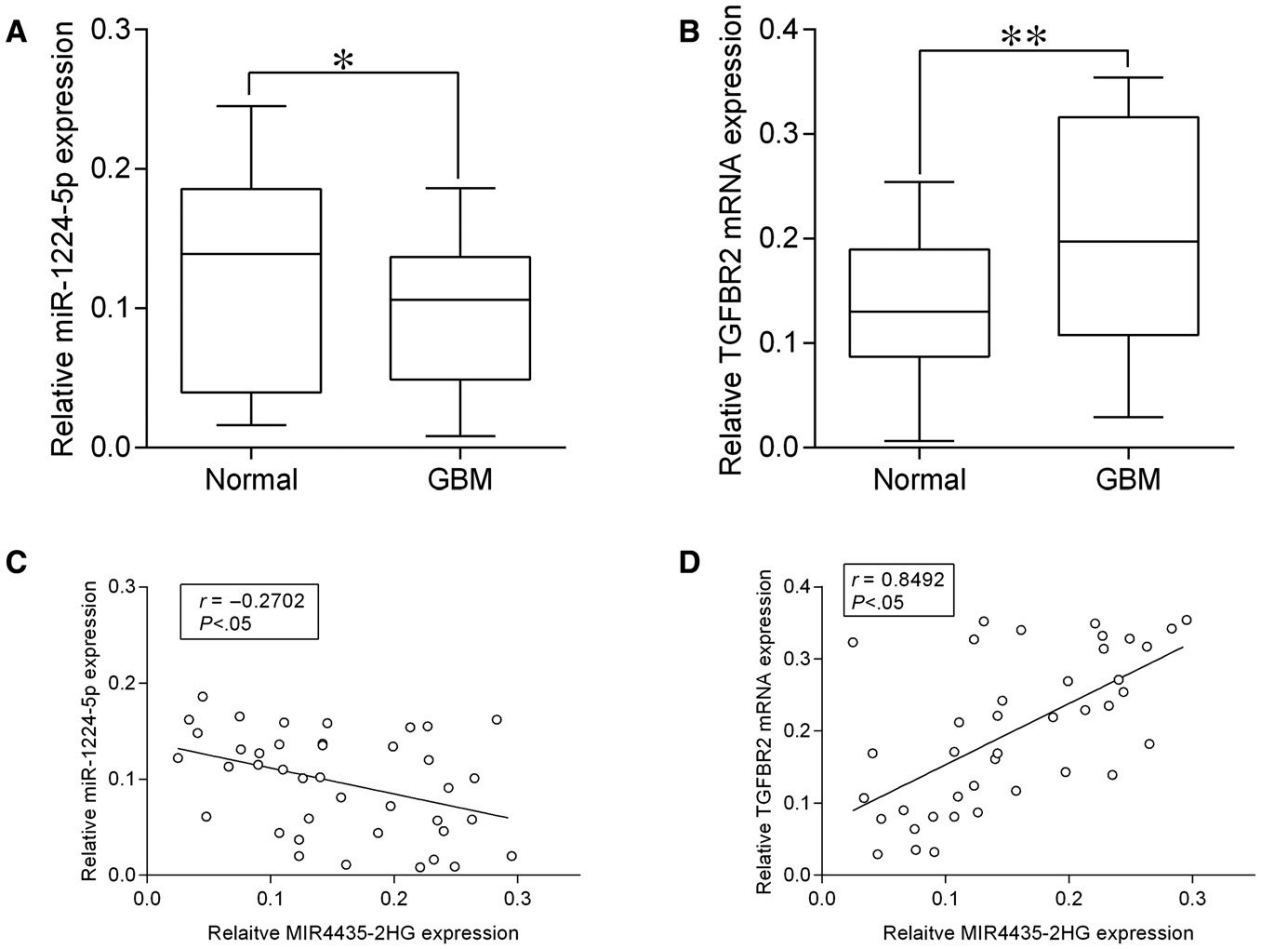
MIR‐1224‐5p and TGFBR2 mRNA expression in clinical tissues. A and B, MiR‐1224‐5p and TGFBR2 in clinical samples were determined by qRT‐PCR, and miR‐1224‐4p (A) was down‐regulated in GBM tissues from 40 patients compared to normal adjacent tissues, while TGFBR2 mRNA (B) was up‐regulated in GBM tissues from 40 patients compared to normal adjacent tissues. (C) The correlation between MIR4435‐2HG and miR‐1224‐5p expression levels and (D) the correlation between MIR4435‐2HG and TGFBR2 mRNA expression levels in GBM tissues were analysed by Pearson's correlation test. *P < .05 and **P < .01

The expression of miR‐1224‐5p and TGFBR2 in clinical tissues was verified with qRT‐PCR. As shown in Figure 7A,B, miR‐1224‐5p was down‐regulated in GBM tissues from 40 patients compared to normal adjacent tissues, while TGFBR2 mRNA was up‐regulated in GBM tissues from 40 patients compared to normal adjacent tissues (Figure 7A,B).Moreover, the MIR4435‐2HG expression level was inversely correlated with miR‐1224‐5p expression level, but was positively correlated with TGFBR2 mRNA expression level in the GBM tissues (Figure 7C,D).

## 4 DISCUSSION

In the present study, to explore the clinical relevance of MIR4435‐2HG in GBM, we firstly derived the MIR4435‐2HG data for GBM from GEPIA database and found that MIR4435‐2HG was up‐regulated in GBM tissues and high MIR4435‐2HG expression was associated with shorter overall survival of GBM patients. Experimentally, the up‐regulation of MIR4435‐2HG was further confirmed in the collected GBM tissues and GBM cell lines. In vitro cell studies and in vivo animal studies presented that knockdown of MIR4435‐2HG resulted in the inhibition of GBM cell proliferation and invasion and in vivo tumour growth, while MIR4435‐2HG overexpression driven GBM progression. Furthermore, MIR44435‐2HG was found to sponge miR‐1224‐5p and suppressed miR‐1224‐5p expression; and overexpression of miR‐1224‐5p attenuated the enhancement in GBM cell proliferation and invasion induced by MIR4435‐2HG overexpression. In a subsequent study, miR‐1224‐5p was found to target TGFBR2 and repressed TGFBR2 expression, and in vitro assays showed that miR‐1224‐5p exerted tumour‐suppressive effects via targeting TGFBR2. More importantly, TGFRB2 knockdown antagonized hyper‐proliferation and invasion of GBM cells with MIR4435‐2HG overexpression. Clinically, the down‐regulation of miR‐1224‐5p and up‐regulation of TGFBR2 were verified in the GBM clinical samples. Taken together, the present study suggests the oncogenic role of MIR4435‐2HG in GBM and underlies the key function of MIR4435‐2HG‐driven GBM progression.

The role of MIR4435‐2HG in cancer progression has been elucidated in several investigations. MIR4435‐3HG was found to be enriched in human gastric cancer tissues and elevated in plasma from these patients.^19^, ^21^ Ouyang et al, showed that up‐regulation of MIR4435‐2HG was associated poor prognosis of colorectal cancer patients and the involvement of MIR4435‐2HG in the colorectal cancer development may be related with p38/mitogen‐activated protein kinases and vascular endothelial growth factor pathways.^18^ MIR4435‐2HG was highly expressed in lung cancer tissues and driven the progression of lung cancer via enhancing the β‐catenin signalling.^20^ In hepatocellular carcinoma, MIR4435‐2HG was up‐regulated in the tumour tissues and exerted the oncogenic actions in hepatocellular carcinoma cells via modulating miR‐487a expression.^17^ In our study, we consistently showed that MIR4435‐2HG was up‐regulated in GBM tissues and cell lines; knockdown of MIR4435‐2HG lead to a decreased activity in GBM cell proliferation and invasion and in vivo tumour growth, while MIR4435‐2HG overexpression caused the opposite effects, suggesting oncogenic role of MIR4435‐2HG in GBM.

The lncRNAs have been well‐studied for mechanistic actions by interacting with different miRNAs, and this action usually leads to miRNA expression repression.^22^ In this study, starBase online prediction tool was utilized and revealed that miR‐1224‐5p had a binding site for MIR4435‐2HG, which was verified by luciferase reporter assay. Additionally, MIR4435‐2HG suppressed the miR‐1224‐5p expression in U87 cells. MiR‐1224‐5p was identified as tumour suppressor in several types of cancers. MiRNA profiling results showed that miR‐1224‐5p was down‐regulated in lung cancer tissues.^23^ Down‐regulation of miR‐1224‐5p was associated with poor overall survival of patients with metastatic colorectal cancer.^24^ In the aspect of lncRNA‐miRNA interaction, the miR‐1224‐5p suppressed melanoma progression and was repressed by lncRNA zinc finger E‐box‐binding homeobox 2 antisense RNA 1.^25^ Additionally, miR‐1224‐5p was tumour‐suppressive in bladder cancer and was negatively regulated by circular RNA hsa_circ_0075828.^26^ In gliomas, miR‐1224‐5p level was decreased in GBM when compared to low‐grade gliomas, and miR‐1224‐5p overexpression suppressed GBM cell proliferation and induced apoptosis via targeting cAMP responsive element binding protein 1.^27^ Consistently, we showed that miR‐1224‐5p overexpression inhibited U87 cell proliferation and invasion and counteracted the GBM progression potentiated by MIR4435‐2HG, suggesting the MIR4435‐2HG‐mediated GBM progression involved in the regulation of miR‐1224‐5p.

To have a deeper understanding into the regulatory network of MIR4435‐2HG in GBM, we utilized starBase tool to further predict the targets of miR‐1224‐5p, and identified TGFBR2 as the target of miR‐1224‐5p. The luciferase reporter assay confirmed the interaction between miR‐1224‐5p and TGFBR2 3’UTR, while in vitro assays showed that TGFBR2 expression was repressed by miR‐1224‐5p overexpression while being promoted by MIR4435‐2HG. TGFBR2 is a key receptor in mediating the tumour growth factor‐beta signal propagation, and knockdown of TGFBR2 was effective in suppressing GBM invasion via a tumour growth factor‐beta‐dependent manner.^28^ In addition, TGFBR2 has also been identified as a novel regulator of GBM stemness,^29^ which may be related to the platelet‐derived growth factor receptor inhibitor resistance in GBM treatment.^30^ TGFBR2 exerted the tumour‐enhancing effects via targeting different downstream mediators such as inhibitor of DNA binding,^31^ Smad2/3 ^32^ and p53 ^33^ in other types of cancers. Moreover, several miRNAs including miR‐181c,^34^ miR‐373 ^35^ and miR‐502c ^36^ suppressed GBM progression via the inhibition of TGFBR2 expression. In the present study, TGFBR2 overexpression attenuated the inhibitory effects of miR‐1224‐5p on GBM progression, while TGFBR2 knockdown counteracted the enhanced GBM progression caused by MIR4435‐2HG overexpression. More importantly, the clinical data showed the down‐regulation of miR‐1224‐5p and up‐regulation of TGFBR2 in GBM tissues. Collectively, the evidence suggests that MIR4435‐2HG promotes GBM progression via the miR‐1224‐5p/TGFBR2 signalling.

To summarize, we showed the up‐regulation of MIR4435‐2HG in GBM tissues and cell lines and uncovered that MIR‐4435‐2HG promoted GBM cell proliferation and invasion via regulating TGFBR2 expression by sponging miR‐1224‐5p. Our study underlies the key function of MIR4435‐2HG‐driven GBM progression and brings forth MIR4435‐2HG as a therapeutic target for this malignant tumour. The study has several limitations. The prognostic role of MIR4435‐2HG should be determined by examining the relationship between MIR4435‐2HG expression level and the survival status of the recruited patients. In addition, studies may further employ the patient‐derived xenograft model of GBM to verify the in vivo effects of MIR4435‐2HG on the GBM progression.

## 5 FUNDING INFOMRATION

This study was supported by the Science, Technology & Innovation Commission of Shenzhen Municipality (No. JCYJ20180305180540801), the Key Scientific Research Project for Young People in Shenzhen Municipal People's Hospital (No. SYKYPY201931) and the General Program of National Natural Science Foundation of China (No. 81572486).

## CONFLICT OF INTEREST

None.

## AUTHOR CONTRIBUTIONS

HX and JM participated in the design of the study and performed the experiments. ZL, BZ and PZ performed the data analysis. HX, PZ, WW, and HZ participated in the development of the experimental design and drafted the manuscript. HX and JM were major contributors to the design of this study and also revised the manuscript. All authors read and approved the final manuscript.

## Supporting information

Figure S1

## Data Availability

The datasets used and/or analysed in this study are available from the corresponding author upon reasonable request.
